# Robotic versus Electromagnetic Bronchoscopy for Pulmonary LesIon AssessmeNT: the RELIANT pragmatic randomized trial

**DOI:** 10.21203/rs.3.rs-3222369/v1

**Published:** 2023-08-28

**Authors:** Rafael Paez, Robert J Lentz, Cristina Salmon, Justin K Siemann, See-Wei Low, Jonathan D Casey, Heidi Chen, Sheau-Chiann Chen, Sameer Avasarala, Samira Shojaee, Otis B Rickman, Christopher J Lindsell, Cheryl L Gatto, Todd W Rice, Fabien Maldonado

**Affiliations:** Vanderbilt University Medical Center; Vanderbilt University Medical Center; Vanderbilt University Medical Center; Vanderbilt University Medical Center; Cleveland Clinic; Vanderbilt University Medical Center; Vanderbilt University Medical Center; Vanderbilt University Medical Center; Case Western Reserve University Hospital: University Hospitals; Vanderbilt University Medical Center; Vanderbilt University Medical Center; Vanderbilt University Medical Center; Vanderbilt University Medical Center; Vanderbilt University Medical Center; Vanderbilt University Medical Center

**Keywords:** electromagnetic navigational bronchoscopy, robotic-assisted bronchoscopy, diagnostic yield, peripheral pulmonary lesion, pragmatic trial

## Abstract

**Background:**

Robotic assisted bronchoscopy has recently emerged as an alternative to electromagnetic navigational bronchoscopy for the evaluation of peripheral pulmonary lesions. While robotic assisted bronchoscopy is proposed to have several advantages, such as an easier learning curve, it is unclear if it has comparable diagnostic utility as electromagnetic navigational bronchoscopy.

**Methods:**

**R**obotic versus **E**lectromagnetic Bronchoscopy for Pulmonary **L**es**I**on **A**ssessme**NT** (RELIANT) is an investigator-initiated, single-center, open label, noninferiority, cluster randomized controlled trial conducted in two operating rooms at Vanderbilt University Medical Center. Each operating room is assigned to either robotic assisted or electromagnetic navigational bronchoscopy each morning, with each OR day considered one cluster. All patients undergoing diagnostic bronchoscopy for evaluation of a peripheral pulmonary lesion in one of the two operating rooms are eligible. Schedulers, patients and proceduralists are blinded to daily group allocations until randomization is revealed for each operating room each morning. The primary endpoint is the diagnostic yield defined as the proportion of cases yielding lesional tissue. Secondary and safety endpoints include procedure duration and procedural complications. Enrolment began on March 6, 2023, and will continue until 202 clusters have been accrued, with expected enrolment of approximately 400 patients by the time of completion in March of 2024.

**Discussion:**

RELIANT is a pragmatic randomized controlled trial that will compare the diagnostic yield of the two most commonly used bronchoscopic approaches for sampling peripheral pulmonary lesions. This will be the first known cluster randomized pragmatic trial in the interventional pulmonology field and the first randomized controlled trial of robotic assisted bronchoscopy.

**Trial registration:**

ClinicalTrials.gov registration (NCT05705544) on January 30, 2023.

## Introduction

### Background and rationale {6a}

Each year, millions of patients are diagnosed with indeterminate peripheral pulmonary lesions (PPLs) and undergo bronchoscopy for further evaluation (1,2). Advanced navigational bronchoscopy systems are often used to accurately reach small peripheral lesions (3,4). Historically, this has mainly consisted of electromagnetic navigational bronchoscopy (ENB). Recently, robotic assisted bronchoscopy (RAB) has emerged as a promising alternative technique. ENB and RAB are routinely used but little comparative data exist on the relative performance of these competing technologies. As such, they are currently used based on local preferences and availability.

Electromagnetic navigational bronchoscopy uses low frequency waves to track a sensor within an electromagnetic field to guide navigation to the lesion of interest (3). The largest prospective multicenter study assessing ENB diagnostic performance and safety reported a diagnostic yield of 67.8% and a complication rate of 4.7% (5). In recent years, digital tomosynthesis, which provides three-dimensional intraprocedural imaging and allows the operator to update the location of the lesion, has been integrated with ENB resulting to diagnostic yield estimates of 75–83% (6–8).

The Food and Drug Administration (FDA) recently cleared a novel RAB platform, the ION^™^ endoluminal system (Intuitive Surgical, Inc., Sunnyvale, CA), which uses a different approach to reach peripheral pulmonary lesions. RAB uses shape sensing technology to track the position of the catheter within the airways. This approach is believed to provide improved stability and movement precision compared to more traditional bronchoscopes. RAB has been rapidly adopted in some settings, primarily because it is believed to be easier to master than ENB. Further, it is believed that the increased stability and precision for fine distal adjustments may become useful for future applications such as bronchoscopic ablation.

Both RAB and ENB were cleared via the 510(k) pathway which does not require a demonstration of improved patient outcomes prior to commercialization, provided safety and effectiveness appear similar to currently marketed device(s). Early comparative data suggest that the diagnostic yield of RAB approaches that of ENB (9), but high-quality data from randomized controlled trials are needed to confirm these preliminary findings. Additionally, both platforms are substantial capital purchases, each costing hundreds of thousands of dollars. Hence, it is also important for health care systems to have access to high quality data when considering device purchases. Since market release, few studies (mostly single center experiences) have been published suggesting the diagnostic yield of RAB is comparable to that of ENB (10–13). The only study to date comparing these technologies is a single center retrospective study which reported similar diagnostic yield (RAB 77% and ENB 80%) and complication rates (9), but selection bias and other confounders limit definitive interpretation.

Both RAB and EMN are available at Vanderbilt University Medical Center and are used interchangeably in the two operating rooms dedicated to interventional pulmonary procedures. Due to the set-up time required and patient workflow, each device is set up in one operating room and used for all patients undergoing biopsy in that room on that day. Patients are scheduled based on operating room availability without any consideration for the device that will be used. We leveraged this variation in clinical practice to design this pragmatic, randomized controlled study to test the hypothesis that the diagnostic yield of RAB is not inferior to ENB in patients undergoing bronchoscopic biopsy of a peripheral pulmonary lesion.

### Objectives {7}

Our primary objective is to compare the diagnostic yield of the ION^™^ Endoluminal System (shape sensing robotic assisted bronchoscopy - ssRAB) to the ILLUMISITE^™^ Platform (electromagnetic navigational bronchoscopy with integrated digital tomosynthesis – DT-ENB) in patients undergoing bronchoscopy for peripheral pulmonary lesion evaluation. Our secondary objective is to compare the rate of complications, procedure time, radiation exposure, diagnostic accuracy at 12 months, and need for additional procedures between these two bronchoscopic techniques.

### Trial design {8}

RELIANT is an investigator-initiated, single-center, open label, pragmatic, non-inferiority, cluster randomized controlled trial. RELIANT compares the diagnostic yield of ssRAB to that of DT-ENB in patients with a peripheral pulmonary lesion requiring navigational bronchoscopic sampling. This study is embedded within usual clinical workflow and has broad eligibility criteria to maximize generalizability (14). The only aspect of clinical care influenced by the protocol is that the navigation platform set-up in each OR each day is randomly rather than arbitrarily allocated.

The cluster level design of the trial was chosen based on how the technologies are used in routine clinical care. While both ssRAB and DT-ENB are mobile, they require substantial time and effort to set up. At VUMC, bronchoscopic biopsies for peripheral pulmonary lesions are performed concurrently in two operating rooms, each using one of the two navigational platforms, which are set up in each room and used for every patient undergoing biopsy in that room on that day. Once set up, logistical aspects of patient workflow prevent moving the platforms from one OR to the other. As part of our usual practice, patients are arbitrarily assigned to a given OR by a scheduler based on schedule availability, not based on specific patient characteristics or provider request. In this way, patients are randomly assigned to a specific OR well in advance of navigation platform allocation, which takes place on the morning of the procedure. This study has been approved by the Vanderbilt University Medical Center Institutional Review Board (IRB 221255). The trial was registered on ClinicalTrials.gov (NCT05705544) prior to the study opening.

## Methods: Participants, interventions and outcomes

### Study setting {9}

RELIANT is being conducted in two operating rooms at Vanderbilt University Medical Center (VUMC), an academic medical center in Nashville, Tennessee, USA.

### Eligibility criteria {10}

Patients will be eligible for inclusion if they are 1) ≥ 18 years of age at the time of bronchoscopy and 2) scheduled for navigational bronchoscopy for the evaluation of a peripheral pulmonary lesion. Patients will be excluded if they are enrolled in another trial which requires one specific navigational platform or if they decline to participate.

### Who will take informed consent? {26a}

Research informed consent will be obtained by the proceduralist or a member of the study team at the time of clinical informed consent for the procedure.

### Additional consent provisions for collection and use of participant data and biological specimens {26b}

Not applicable.

### Interventions

#### Explanation for the choice of comparators {6b}

ssRAB and DT-ENB are the two most widely used navigational bronchoscopy platforms for sampling peripheral pulmonary lesions. Observational comparative data suggest that ssRAB has a diagnostic yield and complication rates that approach those of DT-ENB and may be easier to learn (9). However, high quality comparative data from randomized controlled trials are needed to confirm these findings.

#### Intervention description {11a}

Patients assigned to the ssRAB allocated OR will undergo bronchoscopy with navigation via ssRAB. Patients assigned to the DT-ENB allocated OR will undergo bronchoscopy with navigation via DT-ENB. The study protocol only determines the approach to bronchoscopy, ssRAB or DT-ENB, other clinical decisions will be at the discretion of the bronchoscopist per usual care.

Bronchoscopies with both platforms will follow our routine clinical protocol, which has been previously described (6,7,9). Bronchoscopies are performed under general anesthesia with neuromuscular blockade. Radial endobronchial ultrasound is available for all procedures. Mobile cone beam CT (mCBCT) is available and used at the proceduralist’s discretion. Biopsies are obtained using transbronchial needles, biopsy forceps, cryoprobes, and/or other sampling devices at the discretion of the bronchoscopist. Rapid on-site evaluation (ROSE) is performed in most cases to assess specimen adequacy. All patients recover based on the usual VUMC standard of care, which includes two hours of monitoring in the recovery area. Imaging to assess for possible pneumothorax is obtained if clinically indicated at the discretion of the proceduralist. Post procedure, patients will be managed and followed per usual care. For patients not diagnosed with malignancy, we will review their interval chest CT scans to assess the target lesion for progression, regression, or stability for up to 12 months.

#### Criteria for discontinuing or modifying allocated interventions {11b}

There is no established situation or condition in which ssRAB or DT-ENB is known to be superior. However, in the unlikely scenario that during the conduct of this trial such evidence becomes available, the treating physician would be allowed to modify the allocation as would be expected for usual care. Additionally, if the assigned platform is not functioning or defective, the treating clinician is allowed to use whichever navigational platform is needed for patient care.

#### Strategies to improve adherence to interventions {11c}

Research staff will prospectively monitor to ensure that randomization is used to determine the approach to navigation in both operating rooms throughout the study period, to confirm that all patients receive the strategy to which they are initially assigned, and to confirm that the device assigned to each room and the patients assigned to each room are not changed following randomization. The only threat to adherence would be if the assigned platform is unavailable or non-functional in which case the proceduralist would use the alternative device as needed for patient care, and the patient would be included in the intention-to-treat analysis as a cross-over.

#### Relevant concomitant care permitted or prohibited during the trial {11d}

The only part of the procedure controlled by the study is the bronchoscopy platform. No clinical interventions are prohibited or discouraged by the study.

#### Provisions for post-trial care {30}

No additional ancillary care or follow up beyond what is done for routine clinical care is planned for the purpose of this trial. While rare, complications are expected as part of these procedures and well-documented in the literature (5–13,15). There will be no compensation for participating in the trials or for complications incurred as these are expected as part of routine care.

#### Outcomes {12}

##### Primary Outcome

The primary endpoint will be diagnostic yield, defined as the proportion of procedures that results in acquisition of lesional tissue.

Lesional tissue is defined by the presence of pathological findings that readily explain the presence of a pulmonary lesion. The following common pathological findings are pre-specified:

- Malignancy
- Specific benign pathologic finding including
  - Organizing pneumonia
  - Frank purulence/robust neutrophilic inflammation
  - Granulomatous inflammation
  - Other specific benign findings such as hamartoma, amyloidoma or other uncommon causes of PPLs with distinctive pathological patterns.

Biopsies not meeting any of the above lesional pathological criteria will be adjudicated as not meeting the primary outcome (not being “diagnostic”), including biopsies with normal lung parenchyma or airway components on biopsy, atypia not diagnostic of malignancy, or non-specific inflammation. A blinded panel will review all non-malignant biopsies at the end of accrual to confirm specific benign or non-diagnostic findings on biopsy. Procedures will be adjudicated as not meeting the primary outcome if the procedure starts but biopsies are not obtained (due to failure to navigate to the lesion, or complication, or equipment failure). A procedure will be considered started at induction of general anesthesia.

Biopsies obtained without the use of DT-ENB or ssRAB (e.g., sampling of central lymph nodes using the linear endobronchial ultrasound bronchoscope) will not be included in the diagnostic yield calculations. In case of repeat bronchoscopies, only the index bronchoscopy will be included in the diagnostic yield calculation.

Diagnostic yield is one of the most commonly reported outcomes when evaluating advanced bronchoscopic methods (2,5–13,15). We have chosen the most conservative definition of diagnostic yield, consistent with our prior publications. (6–9).

##### Secondary, Safety and Exploratory Outcomes

The sole prespecified secondary outcome will be the duration of the navigation procedure (in minutes), defined as time from the start of airway registration to the removal of the bronchoscope after completion of navigation procedures.

Safety outcomes will focus on procedural complications within 7 days of the procedure including:

- Pneumothorax
- Bronchopulmonary haemorrhage
- Respiratory failure
- Anesthesia complications

Exploratory clinical outcomes are:

- Radiation exposure, defined as radiation dose delivered to the patient during the study bronchoscopy, recorded as a dose area product (mGy/cm2)
- Additional diagnostic procedures, defined as any diagnostic procedure performed after the study bronchoscopy which targets the same peripheral lesion for diagnostic purposes (including repeat bronchoscopy, transthoracic needle biopsy, or surgical lung biopsy) between completion of the index procedure and 12 months. Repeat biopsies of lesions determined to be malignant by study bronchoscopy which are performed specifically to obtain additional tissue for further testing but that does not change the malignant diagnosis, will not be considered additional diagnostic procedures.
- Diagnostic accuracy at 12 months post-biopsy, defined as the number of true positive lesions (malignant) plus true negative lesions (specific benign diagnosis) with no evidence of malignancy at 12-month follow-up (no interval biopsy diagnostic of malignancy, regression on CT or stable size with no plan for repeat diagnostic procedure), divided by the total number of biopsied lesions.

#### Participant timeline {13}

#### Sample size {14}

The diagnostic yield of DT-ENB varies widely in the literature based on the definition (2, 5–8, 15). Based on data from prior studies using a similar diagnostic yield definition as this trial, we estimate the diagnostic yield of DT-ENB to be 80% (6–9). The diagnostic yield of ssRAB has not been fully elucidated, but published data suggest an overall diagnostic yield approaching 80% as well (9–13). Assuming the diagnostic yield for DT-ENB is 80%, with a non-inferiority margin set at 10%, cluster size of 2, and no intracluster correlation, 202 clusters (OR-days) would provide an 80% power to conclude noninferiority at a one-sided type I error rate of 5%. The noninferiority margin was chosen based on what would be considered a clinically significant difference that would favor ENB over RAB, despite RAB’s better learning curve and enhanced stability. We assumed no intracluster correlation given the very small cluster size and the low likelihood that patients in each cluster will be significantly different. Based on an average of 2 cases per day, we anticipate that approximately 400 patients will be enrolled in the 202 clusters of this trial.

#### Recruitment {15}

The Interventional Pulmonology group at Vanderbilt University Medical Center performs over 400 navigational bronchoscopies per year making it one of the highest volume centers in the US. Every patient undergoing navigational bronchoscopic biopsy of PPL will be eligible for inclusion in the study. Given the broad inclusion criteria and the comparison of modalities that patients would experience as part of routine clinical care outside of research, we expect that the number of patients who decline to participate will be small.

### Assignment of interventions: allocation

#### Sequence generation {16a}

Parallel cluster randomization is used for this study given impracticability of patient-level 1:1 randomization. The ssRAB and DT-ENB platforms are randomly allocated based on operating room day. A biostatistician not involved in patient care generated the randomization sequence using R programming language in randomly permuted blocks of size 2 and 4, stratified by day of the week. Stratification by day of the week was added to minimize imbalances that could arise due to differences in the amount of time each provider spends in each operating room (e.g., due to vacation). One randomization sequence was generated for each day, and a mirror sequence with the opposite allocation was assigned to the second operating room on days where both operating rooms are scheduled to perform bronchoscopy for peripheral pulmonary lesions. On these days, a single envelope contains the specific allocation assigned to each room.

#### Concealment mechanism {16b}

Allocation is concealed in sealed, opaque envelopes, clearly marked with the day of the week, and are opened by the OR staff each morning on days with navigational bronchoscopy cases scheduled. The envelopes were prepared by support staff not involved in patient recruitment or scheduling. The envelopes are located in one of the operating rooms in a clearly marked, secured box.

#### Implementation {16c}

The allocation sequence was generated by a statistician not involved in clinical care. The participants are enrolled by the study personnel, including interventional pulmonary attendings, fellows, and/or advanced practice providers, who obtain consent for both the navigational bronchoscopic procedure and the collection of data and use for research. Participants are assigned to interventions according to the day their bronchoscopic procedure is scheduled by the blinded schedulers.

### Assignment of interventions: Blinding

#### Who will be blinded {17a}

SPIRIT guidance: Who will be blinded after assignment to interventions (eg, trial participants, care providers, outcome assessors, data analysts), and how.

Patients are scheduled for their procedure days in advance by a blinded scheduler without any knowledge of what platform the OR will be randomized to for the scheduled day of the procedure. It is not possible to blind the bronchoscopist or the patient to the platform used the day of the procedure, as they are both large distinctive-appearing pieces of equipment. However, the patient, the proceduralist, and the scheduler are blinded at the time of scheduling and will remain blinded until the day of the procedure. Additionally, thoracic pathologists are blinded throughout the study, such that allocations will not influence the histopathological interpretation.

#### Procedure for unblinding if needed {17b}

Not applicable.

### Data collection and management

#### Plans for assessment and collection of outcomes {18a}

All data collected in this study are captured as part of routine clinical care. Data will be collected in a Health Insurance Portability and Accountability Act (HIPAA) compliant REDCap database (16). The database is composed of several case report forms (CRFs) that include demographic, radiographic, procedure, complication, histopathology and follow up data. Data will be entered by the proceduralists, and study team members who are trained in use of the CRF. Data collected in the CRFs include but are not limited to: study participants’ demographic and risk factors for lung cancer, nodule size and other nodule characteristics, procedure details including duration, platform and tools used, specific histopathologic findings, radiation dose and any complications that occur during or after the procedure. Follow up data including repeat bronchoscopic biopsy and additional biopsies obtained by alternative means (transthoracic needle biopsy or surgical biopsy) will be collected by review of the electronic health record.

#### Plans to promote participant retention and complete follow-up {18b}

RELIANT is a pragmatic trial embedded in our clinical practice. As such, patients will be followed per usual care at the discretion of the proceduralist. Primary, secondary and safety outcomes will be captured by the end of the procedure or adjudicated within seven days. Follow up for the purpose of this study is only required for two of the exploratory outcomes, diagnostic accuracy at 12 months and additional diagnostic procedures. We, therefore, expect the rate of missingness due to loss to follow-up to be low. Given that the primary purpose of biopsy for peripheral pulmonary lesions is the evaluation of possible malignancy, the vast majority of patients return to their follow up appointments after biopsy. Standard operating procedures are in place at VUMC to ensure patient follow-up, which should also minimize loss to follow-up.

#### Data management {19}

REDCap is a secure, HIPAA compliant, web-based application (16). Only key study personnel approved by the IRB will have access to the database. Data will be entered daily by the proceduralists, and study team members experienced with the database. The database was built with data quality checks in place such as specific data entry restrictions e.g., integer only, ranges, etc. Key data fields have been marked as required and need to be completed before finalizing the CRF.

#### Confidentiality {27}

All patient related information in this study will be entered and stored in a Vanderbilt University Medical Center REDCap database, which requires two factor authentications if accessed from outside of VUMC’s firewall. Research informed consents will be kept in a research binder in a locked file cabinet in a locked office. Only key study personnel approved by the IRB will have access to this database and binder as necessary to conduct the research. Every effort will be made to protect the privacy of research subjects. Subject names and protected health information (PHI) will be kept confidential to the extent possible and as required by applicable laws and regulations. All records and data related to the study will be maintained in secure protected spaces, with access restricted to key study personnel approved by the IRB who (i) need access to the information to fulfill the terms and obligations under the Protocol and (ii) are under the same obligations as study personnel to keep the information confidential. Following publication, a de-identified dataset will be created.

### Plans for collection, laboratory evaluation and storage of biological specimens for genetic or molecular analysis in this trial/future use {33}

Not applicable.

### Statistical methods

#### Statistical methods for primary and secondary outcomes {20a}

##### Descriptive Analysis

To characterize the study sample, baseline demographic and clinical data will be described overall and by group. Categorical variables will be described using frequencies and proportions, and continuous variables will be described using means and standard deviations or medians and interquartile ranges, as appropriate. Missingness will be reported for each variable. At a minimum, the following variables will be described at time of enrollment:

- Age (years)
- Gender (male, female, unknown)
- Race (African American, Asian/Pacific Islander, Caucasian, Multiple, Native American, Other, Unknown)
- Ethnicity (Hispanic, Non-Hispanic, Unknown)
- History of cancer (yes/no)
- Smoking History (current, former, never)
- Body mass index (BMI)
- Lesion characteristics
  - Size (mm)
  - Location (middle vs peripheral)
  - Upper lobe (yes/no)
  - Density (solid, part solid, ground glass opacity)
  - Distance from pleura (cm)
  - Bronchus sign present (yes/no)
- Procedure details
  - Rapid onsite evaluation (yes/no)
  - Operator
  - Digital tomosynthesis (yes/no)
  - Cone beam computed tomography (yes/no)
  - Biopsy tools

We will describe the outcome variables overall and grouped by study arm using the same approach as for the demographic data. Summary statistics and graphical representations may be displayed, and missingness will be reported for each variable. No statistical comparisons between groups will be done for this descriptive analysis.

##### Primary analysis

The primary analysis for the trial will use an intent-to-treat approach. Participants will be evaluated by treatment group as assigned regardless of what was delivered. All eligible participants will be included.

The primary outcome variable (diagnostic yield) will be compared between groups using a generalized linear mixed model with one-sided test. The primary model will be covariate adjusted, including fixed effects for device assignment, lesion size, density, peripheral location, and bronchus sign, and a random effect for operator. Should the model demonstrate signs of overfitting, covariates may be selected based on priority order (device assignment, lesion size, density, peripheral location, bronchus sign, operator).

If non-inferiority is demonstrated, we will proceed with a superiority analysis using the same approach as the non-inferiority analysis, an adjusted generalized linear mixed effects model with the same covariates used in the primary analysis. No adjustment will be made for multiplicity. The superiority analysis would be conducted at a p-value of 0.05.

##### Secondary and exploratory analysis

A sensitivity analysis using a *per protocol* approach will be conducted to analyze participants based on the device used. We will use the same approach as the primary analysis, an adjusted generalized linear mixed effects model using the same covariates.

The secondary outcome, procedure duration, will be compared between study groups using a generalized linear mixed model. If the procedure duration is skewed, alternative models may be pursued, such as a cox regression. The primary model will be covariate adjusted following a similar approach to the analysis of the primary outcome. Analysis of the exploratory endpoints will follow a similar approach.

##### Safety analysis

Procedure complications are expected in usual care, although uncommon. We will report all procedure complications for each device, overall and by type. If event rates exceed 5%, we may proceed with a comparative analysis, which will involve a generalized linear regression model for binary outcomes as specified for the main analysis, with the exception that covariate adjustment may not be possible. The safety analysis dataset will group participants by device used, regardless of assignment.

All model results will be summarized with point estimates and 95% confidence intervals (CIs), which will be emphasized over p-values when reporting the results for secondary outcomes. No adjustments for multiplicity will be made.

#### Interim analyses {21b}

There is no plan for interim analysis and thus no boundaries for early stopping.

#### Methods for additional analyses (e.g. subgroup analyses) {20b}

##### Differential effects

To determine whether differences in outcomes are dependent on baseline characteristics, we will introduce interaction terms into the models developed for the main analysis. Specifically, we will test the interaction between device assignment and the subgrouping variable. Each variable will be tested one by one, such that all main effects but only one interaction term is included at a time. The following putative subgrouping variables are prespecified:

- Nodule size (continuous, treatment effects will be estimated at 1.5cm and 3cm)
- Presence of bronchus sign
- Solid vs subsolid nodule
- Peripheral vs central location – *Peripheral defined as outer 1/3 of chest*

### Methods in analysis to handle protocol non-adherence and any statistical methods to handle missing data {20c}

Missingness on the primary or secondary outcome is not expected due to the proximity of its measurement with the procedure and its integration into clinical documentation. Procedures missing the primary outcomes will be considered not diagnostic. Missing covariates will be imputed using multiple imputation with predictive mean matching. There may be missingness on exploratory outcomes. For missing exploratory outcomes, a complete case analysis will be performed.

#### Plans to give access to the full protocol, participant level-data and statistical code {31c}

The protocol and the statistical analysis plan will be available with the published study manuscript. Deidentified participant-level dataset will be available upon reasonable request and with appropriate IRB approval.

### Oversight and monitoring

#### Composition of the coordinating centre and trial steering committee {5d}

There is no coordinating center or trial steering committee for this single center randomized control trial embedded within usual clinical care.

#### Composition of the data monitoring committee, its role and reporting structure {21a}

The Data and Safety Monitoring Board (DSMB) consists of 3 members with expertise appropriate to the conduct of the study, including thoracic surgery, pulmonary medicine, biostatistics, and clinical trials. The DSMB will evaluate any research related serious adverse events (SAEs) or unanticipated adverse events (AEs). Outcome data may be presented to the DSMB at the DSMB’s request with no plan for interim analyses. Appointment of all members was contingent upon the absence of any conflicts of interest. All the members of the DSMB are voting members. The DSMB developed a charter and reviewed the protocol and appropriate regulatory documents during its first meeting. The DSMB will meet two additional times at approximately 33% and 66% enrollment to review safety event reports in aggregate, and *ad hoc* as needed. The DSMB will have the ability to recommend that the trial end, be modified, or continue unchanged.

#### Adverse event reporting and harms {22}

This pragmatic randomized controlled trial is embedded within usual clinical care and compares two standard of care approaches to biopsy peripheral pulmonary lesions. Clinical adverse events are well documented in the literature and discussed in detail with the patient during the clinical procedure consent. Clinical adverse events are, therefore, considered to be related to the clinical procedure rather than related to the research. Clinical adverse events are systematically collected and reviewed by the DSMB but not individually reported to the IRB. The primary risk of research participation is breach in privacy and confidentiality and many safeguards have been established as detailed previously. Serious unanticipated adverse events will be reported to the DSMB and IRB per current institutional standards.

#### Frequency and plans for auditing trial conduct {23}

There is no plan for trial auditing.

### Plans for communicating important protocol amendments to relevant parties (e.g. trial participants, ethical committees) {25}

We anticipate no major changes to the protocol during the conduct of this study. If needed, however, any changes to the protocol will be made in the form of a written amendment and must be approved by the IRB prior to implementation. Protocol changes to eliminate an immediate hazard to a trial patient may be implemented by the investigator immediately. The investigator must then immediately inform the IRB and DSMB. All proceduralists are co-investigators and actively involved in this study, as such, any important modification will be communicated in person or via email.

### Dissemination plans {31a}

Any manuscript or releases resulting from the collaborative research must be approved by the investigators and will be circulated to applicable participating investigators prior to submission for publication or presentation. A publication plan consistent with the International Committee of Medical Journal Editors (ICMJE) will be created prior to analysis and publication of any data. All data will be made available to authors as required. The publication of sub-studies and post-hoc analyses will not precede the primary publication. Publication of results will be determined by the investigators. All authors are expected to disclose financial relationships or affiliations that could be considered conflicts of interest per journal or medical society requirements.

## Discussion

RELIANT is the first randomized trial comparing two commonly used advanced bronchoscopy platforms for sampling peripheral pulmonary lesions. Furthermore, it is the first pragmatic trial comparing routinely used bronchoscopic devices in the Interventional Pulmonology field. Given how commonly advanced bronchoscopy is pursued in the United States, this study has the potential to immediately inform patient care and fill this important knowledge gap. To date, only a few randomized controlled studies have been conducted comparing different bronchoscopic modalities for sampling peripheral pulmonary lesions. However, these have been conducted using more traditional designs, with more stringent eligibility criteria and evaluated older technologies (17–20).

We considered several possible designs for this trial and weighted their potential advantages and disadvantages. Most importantly, the study was designed in a pragmatic manner in order to minimize disruptions in workflow and allow for rapid accrual to produce results in a timely fashion. While cluster randomized trials may have lower precision and higher risk of bias, it was the best design to answer our question and meet our other key goals: pragmatic trial with broad eligibility criteria, embedded within the clinical workflow to minimize disturbances, and pragmatic endpoint that would allow for fast adjudication. The RELIANT study design has the potential to overcome the challenge of generating meaningful data at a time when technology evolves too quickly for evaluation via conventional study designs and when devices are cleared for clinical use without extensive comparative data.

We selected diagnostic yield as our primary endpoint for several reasons: 1) immediate adjudication allows for quick generation of clinically relevant data that can inform patient care while mitigating the burdens of long term follow up, 2) a pragmatic patient-centered endpoint as the data are available clinically and used to inform patient care and 3) limited difference between diagnostic yield and diagnostic accuracy when a conservative definition criterion is used. The secondary and exploratory outcomes are also important patient centered outcomes such as duration of procedure, complications, and radiation exposure.

There are several potential limitations of this study. First, this is a single center study with very experienced operators, thus generalizability may be limited to this setting. However, the single center design mitigates the effect of experience as potential confounder as all the supervising proceduralists are experienced using both platforms. We are comparing these two technologies in their current versions, but these may change during the conduct of this trial, which highlights the importance of our study design capable of generating data in a timely manner. We decided not to modify any aspects of the procedures other than platform allocation. This could potentially affect the results if certain aspects of the procedure influence the primary outcome e.g., tools used. However, we elected for this option rather than protocolize the procedure to minimize disruptions to routine patient care and maximize pragmatism, real-world setting, and generalizability. Allocation concealment is a potential source of bias in cluster randomized trials. While this is certainly a limitation, we have minimized any possible bias by opening the allocation envelope on the day of the procedure, rendering it impossible for the scheduler, proceduralist or patient to know in advance which platform will be set up for the day in a given operating room. In addition, in our routine practice, patients are not allowed to change operating rooms on days when both platforms are in use in separate rooms. Furthermore, if a patient is randomized and enrolled the procedure will be included in the intention to treat analysis regardless of the intervention received. Finally, pathologists are blinded to the allocation as specimens do not contain information on the device used.

In summary, RELIANT is a single center, open label, noninferiority, pragmatic cluster randomized controlled trial comparing the diagnostic yield of two of the most commonly used platforms for sampling peripheral pulmonary lesions, DT-ENB and ssRAB.

### Trial status

The trial opened on March 6, 2023, and is expected to complete enrollment in March of 2024.

Protocol version: 3

Date: 4/23/2023

## Figures and Tables

**Figure 1:**
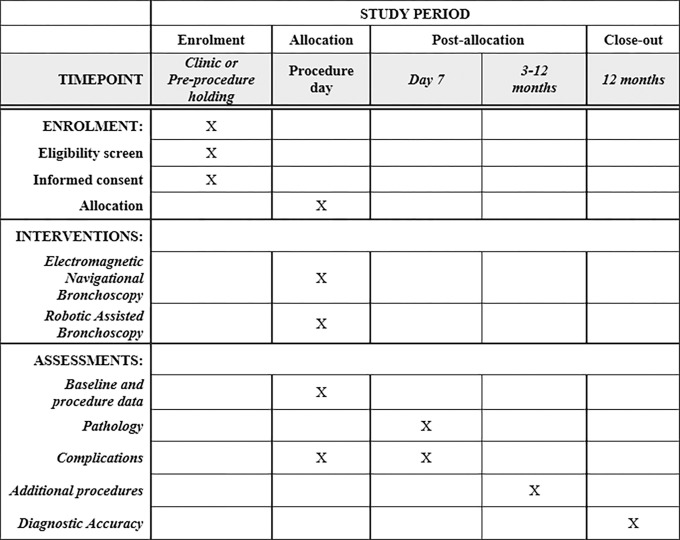
Standard Protocol Items: Recommendation for Interventional Trials. RELIANT enrolment, intervention, and assessment.

## Data Availability

Deidentified participant-level dataset will be available 3 months after the final results are published for reasonable request and appropriate IRB approval. The request can be sent to the corresponding author, Fabien Maldonado or Rafael Paez.

## List of Learning Healthcare System Platform Investigators

Vanderbilt University Medical Center and Vanderbilt University, Nashville, TN

Gordon R. Bernard, Robert S. Dittus, Shon Dwyer, Peter J. Embi, Chad Fitzgerald, Robert E. Freundlich, Cheryl L. Gatto*, Frank E. Harrell Jr., Paul A. Harris, Tina Hartert, Jim Hayman, Catherine H. Ivory, Ruth Kleinpell, Sunil Kripalani, Lee Ann Liska, Patrick Luther, Jay Morrison, Thomas Nantais, Jill M. Pulley, Kris Rehm, Todd W. Rice*, Russell L. Rothman, Patti Runyan, Wesley H. Self, Matthew W. Semler, Robin Steaban, Cosby A. Stone Jr., Philip D. Walker, Consuelo H. Wilkins, Adam Wright, and Autumn D. Zuckerman.

* Denotes members of the RELIANT Trial Study Team

## Abbreviations

ENB: Electromagnetic navigation bronchoscopy
DT-ENB: Electromagnetic navigation bronchoscopy with integrated digital tomosynthesis
RAB: Robotic assisted bronchoscopy
ssRAB: Shape sensing robotic assisted bronchoscopy
PPL: Peripheral pulmonary lesion
EBUS: Endobronchial Ultrasound
OR: Operating room
RELIANT: **R**obotic versus **E**lectromagneticBronchoscopy forPulmonary **L**es**I**on **A**ssessme**N**T
FDA: Food and Drug Administration
CT: Computed Tomography
LG: Locatable Guide
VUMC: Vanderbilt University Medical Center
PHI: Protected Health Information
DSMB: Data and Safety Monitoring Board
CRF: Case Report Form
IRB: Institutional Review Board
ICMJE: International Committee of Medical Journal Editors
HIPAA: Health Insurance Portability and Accountability Act
